# Health tips in newspapers: a matter of concern

**DOI:** 10.11604/pamj.2020.37.52.19997

**Published:** 2020-09-14

**Authors:** Pushparaja Shetty

**Affiliations:** 1 Department of Oral Pathology and Maxillofacial Pathology and Oral Microbiology, AB Shetty Memorial Institute of Dental Sciences, Nitte (Deemed to be University), Deralakatte, Mangalore, India

**Keywords:** Newspapers, health tips, health reporting

## Abstract

Newspapers are a significant source of information to the general public as it reaches quite large numbers. Many readers make an essential health decision based on the information provided by the newspaper. Evidence suggests that quality of health reporting in the newspaper is poor. Most of the health tips in the newspaper may lead to self-medication, which is most dangerous. It is the responsibility of the newspaper to provide health information with scientific evidence and readers to be cautious before making any health-related decision based on newspaper information without consulting experts.

## Opinion

It is a well-known fact that newspapers are a significant source of information to the general public as it reaches quite a large number of population. The newspapers had created a positive impact in creating public awareness on various aspect of health mainly against substance abuse, tobacco and various preventive measures of many diseases [1, 2]. There is a high demand and need for accurate, relevant and essential information in the health sector. Many readers make an important health decision based on the information provided by the newspaper [3]. Readers blindly follow the contents without any check. Health tips in the media are common in herbal medicine and supplements. The primary concern of this information is quality. Many newspapers concentrate on over expressing the benefits of certain products/items and undercover the risk involved. Evidence suggests that quality of health reporting in the newspaper is poor [3]. The reliability of the information, sources of study are usually not provided to the readers. Many often, readers are not able to differentiate whether the information provided is from professional experts or otherwise.

Matter influencing readers may be from a preliminary study and information provided from a press release of scientific proceedings [4]. Information may be incomplete and with insufficient scientific evidence and validity. Sometimes, pharmaceutical companies directly or indirectly promote their products through certain articles. Herbal medicine and products are often projected as safest and marketed as natural products; however, excess use of some herbal products may cause kidney and liver damage and also they interfere with other medicines. The adverse effect of many herbal products is neither studied nor reported. Newspaper health tips are more often on nutrition, which is the most complicated subject and presented in the media with many contradicting points. Readers exposed to such information are less likely influenced by health experts. Most of the health tips in the newspaper may lead to self-medication, which is most dangerous. It is the responsibility of the newspaper to provide health information with scientific evidence and readers to be cautious before making any health-related decision based on newspaper information without consulting experts.
